# *Mycobacterium tuberculosis*: Rewiring host cell signaling to promote infection

**DOI:** 10.1002/JLB.4MR0717-277R

**Published:** 2017-12-15

**Authors:** Michael D. Stutz, Michelle P. Clark, Marcel Doerflinger, Marc Pellegrini

**Affiliations:** ^1^ Division of Infection and Immunity The Walter and Eliza Hall Institute of Medical Research Parkville Victoria Australia; ^2^ Department of Medical Biology The University of Melbourne Parkville Victoria Australia

**Keywords:** host‐pathogen interactions, virulence factors, macrophages

## Abstract

The ability of *Mycobacterium tuberculosis* to cause disease hinges upon successfully thwarting the innate defenses of the macrophage host cell. The pathogen's trump card is its armory of virulence factors that throw normal host cell signaling into disarray. This process of subverting the macrophage begins upon entry into the cell, when *M. tuberculosis* actively inhibits the fusion of the bacilli‐laden phagosomes with lysosomes. The pathogen then modulates an array of host signal transduction pathways, which dampens the macrophage's host‐protective cytokine response, while simultaneously adapting host cell metabolism to stimulate lipid body accumulation. *Mycobacterium tuberculosis* also renovates the surface of its innate host cells by altering the expression of key molecules required for full activation of the adaptive immune response. Finally, the pathogen coordinates its exit from the host cell by shifting the balance from the host‐protective apoptotic cell death program toward a lytic form of host cell death. Thus, *M. tuberculosis* exploits its extensive repertoire of virulence factors in order to orchestrate the infection process to facilitate its growth, dissemination, and entry into latency. This review offers critical insights into the most recent advances in our knowledge of how *M. tuberculosis* manipulates host cell signaling. An appreciation of such interactions between the pathogen and host is critical for guiding novel therapies and understanding the factors that lead to the development of active disease in only a subset of exposed individuals.

**Abbreviations**
3HB: D‐3‐hydroxybutyrate
BCG: bacillus Calmette‐Guérin
cGAS: cyclic GMP‐AMP synthase
DC: dendritic cell
ER: endoplasmic reticulum
ESAT‐6: early secreted antigenic target 6
Mce3E: mammalian cell entry protein 3E
STING: stimulator of interferon genes
TAG: triacylglycerol
TNT: tuberculosis necrotizing toxin
V‐ATPase: vacuolar‐H^+^‐ATPase

## 1 INTRODUCTION

*Mycobacterium tuberculosis* has coexisted with mankind for tens of thousands of years, claiming more lives than any other infectious agent. This long history of coevolution with humans has given rise to a pathogen uniquely capable of persisting even in the face of a plethora of host antimicrobial effector mechanisms. *Mycobacterium tuberculosis* thus continues to cause devastating morbidity and mortality, killing 1.8 million people in 2015 alone, and latently infecting an estimated one quarter of the world's population.^1^

*Mycobacterium tuberculosis* is transmitted between hosts by aerosols, which are capable of traveling to distal regions of the lung. The first cells to encounter the mycobacteria are alveolar macrophages, which attempt to eliminate the pathogen through the innate antimicrobial process of phagocytosis. The ability of *M. tuberculosis* to establish a productive infection is entirely contingent on its survival in the macrophage during this early stage. The majority of infected people efficiently restrain its growth, such that the pathogen persists latently in the absence of overt disease. Around 5–15% of infected individuals will develop active life‐threatening disease during their lifetime.^2^ Exposure to immunosuppressive agents greatly increases the risk of developing active disease. The remarkable capacity of *M. tuberculosis* to persist even in the context of a fully immunocompetent host bespeaks its aptitude at resisting multiple host antimicrobial defenses. *Mycobacterium tuberculosis* lacks many of the classical bacterial virulence factors such as toxins and flagella, which are advantageous to pathogens that must compete with the mucosal microflora in order to colonize the host, but the need for which is obviated for pathogens that target sterile sites deep in the lung.^3^ Instead, *M. tuberculosis* has evolved a cornucopia of refined adaptations to escape immunity and persist within the host. Perhaps most interesting and sophisticated are the mechanisms by which the pathogen systematically disables, stimulates, or reroutes normal host cell signaling pathways to promote its own survival.

Nearly 140 years have passed since Robert Koch identified *M. tuberculosis* as the causative agent of tuberculosis (TB). Despite intense research efforts to understand the complex and dynamic interactions between this pathogen and its host, the process of discovering what makes *M. tuberculosis* such a remarkably successful pathogen continues to the present day. This minireview critically examines the most recent advances in our understanding of how *M. tuberculosis* modulates host signaling, with the goal of highlighting potential avenues for novel therapeutic interventions.

### 1.1 Hijacking phagosomes as a replicative niche

**Figure 1:**
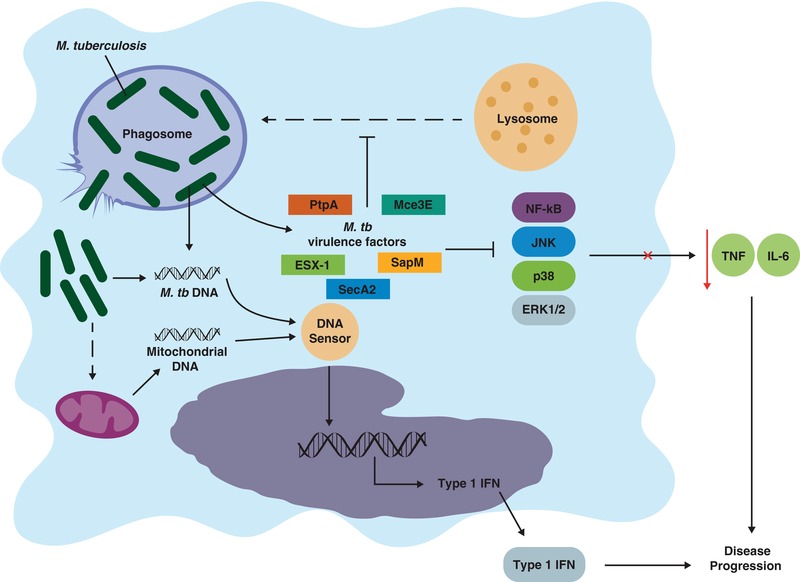
***Mycobacterium tuberculosis* manipulates essential components of the macrophage antimicrobial response**. Mycobacterial virulence factors such as ESX‐1, PtpA, and SecA2 interfere with the ability of lysosomes to kill phagocytosed *M. tuberculosis*, thereby establishing a secure, intracellular replicative niche. The pathogen then disables NF‐κB and MAPK signal transduction pathways, blunting the macrophage's host‐protective cytokine response. However, bacilli may eventually burst from phagosomes to activate host cytosolic DNA sensors, either directly by shedding their own DNA, or indirectly by inducing mitochondrial stress and DNA release. The consequence of this is the transcriptional induction of type I IFNs, which are detrimental to the host

Over 45 years ago, Armstrong and Hart reported the archetypical virulence mechanism of *M. tuberculosis*—the inhibition of phagosome‐lysosome fusion in macrophages.^4^ Later work revealed that macrophages additionally fail to acidify mycobacteria‐laden phagosomes, which was attributed to their inability to recruit host vacuolar‐H^+^‐ATPase (V‐ATPase).^5^ It was proposed that *M. tuberculosis* resists the maturation of phagosomes in order to exploit the organelle as an intracellular replicative niche. Phagosomes normally interact with the endosomal compartment to recruit V‐ATPase, which actively transports protons into the phagosome, generating a potently acidic lumen that is required for subsequent fusion with lysosomes and the activity of the antimicrobial molecules they deliver.^6^ Recent work showed that the secreted mycobacterial protein tyrosine phosphatase PtpA permeates through the phagosome membrane into the cytosol and binds to subunit H of host V‐ATPase.^7^, ^8^ This binding disrupts the tethering of V‐ATPase to the phagosome membrane, and also localizes PtpA in close proximity to its catalytic substrate, vacuolar protein sorting (VPS)33B, which is involved in regulating endocytic membrane fusion. Interference with V‐ATPase recruitment and dephosphorylation of VPS33B by PtpA are both required for the inhibition of phagosome acidification and phagosome‐lysosome fusion.^7^ Interestingly, although deletion of *PtpA* restricts bacterial growth within human THP‐1 cells,^8^ a deletion mutant was not attenuated for growth or virulence during *in vivo* infection of mice.^9^ This discrepancy was attributed to potential species differences affecting the activity of PtpA, such that reduced activity in a particular species would obscure any defect caused by its deletion. However, equally likely is the existence of some degree of functional redundancy between particular mycobacterial virulence factors that only becomes apparent during *in vivo* infection. In fact, numerous virulence factors have been reported to interfere with phagosome‐lysosome fusion *in vitro*, including SecA2, PknG, SapM, components of the ESX‐1 secretion system, and various glycolipids (Fig. 1).^10^, ^11^, ^12^, ^13^, ^14^ The deletion of some of these virulence factors was found to attenuate bacterial growth and disease in animal models.^11^, ^15^ While this could indicate that certain virulence factors play nonredundant roles in inhibiting phagolysosome formation *in vivo*, several other roles have been reported for these factors, some of which are discussed later in this review. Collectively, although the literature supports the inhibition of phagosome maturation as a potent virulence mechanism of *M. tuberculosis*, the reported multifunctional roles of many of the reported effectors of this inhibition makes it difficult to determine the extent to which their phagolysosome‐inhibitory activity alone impacts on overall disease pathogenesis.

### 1.2 The phagosome—a Trojan horse?

Until recently, *M. tuberculosis* was thought to reside within the phagosomal compartment of macrophages for the entire duration of the infection cycle, with release occurring upon lytic cell death. This paradigm shifted, however, with reports describing rupture of the phagosomal membrane and translocation of *M. tuberculosis* to the cytosol.^16^, ^17^, ^18^ This escape from phagosomes appears to be dependent on both ESX‐1 and particular bacterial outer membrane lipids (phthiocerol dimycocerosates).^19^ In 2015, 3 independent studies simultaneously reported the activation of cytosolic DNA sensing pathways resulting from this egress of *M. tuberculosis* from phagosomes.^20^, ^21^, ^22^ The DNA sensor cyclic GMP‐AMP synthase (cGAS) was shown to associate with *M. tuberculosis* DNA in the cytosol to stimulate cyclic GAMP synthesis. This secondary messenger activates stimulator of interferon genes (STING), which drives the STING‐TBK1‐IRF3 signaling pathway, leading to the transcriptional induction of type I IFNs, particularly IFN‐β (Fig. 1). Collectively, the studies showed that deletion of the cGAS‐STING pathway abrogated IFN‐β secretion by macrophages infected *in vitro* with *M. tuberculosis*. However, this *in vitro* phenotype was only partially replicated in mice deficient in either cGAS or STING, which had slightly reduced serum IFN‐β levels but otherwise unaltered inflammatory markers.^20^, ^21^ Furthermore, this reduction did not translate into any defect in resistance to *M. tuberculosis* infection, with *Cgas^−/−^* or *Sting^−/−^* mice harboring similar bacterial numbers in all organs at all time points. One study did, however, report earlier mortality of *Cgas^−/−^* but not *Sting^−/−^* mice compared with wild‐type mice.^21^ This raises two points that require further investigation; first, additional pathways besides cGAS‐STING contribute to *M. tuberculosis* induced IFN‐β production *in vivo*, and second, perhaps cGAS has other functions conferring resistance to chronic infection.

### 1.3 Modulation of type I IFN signaling

Type I IFNs are potent mediators of antiviral immunity, but are often associated with disease progression in bacterial infections. C57BL/6 mice deficient in the type I IFN receptor (IFNAR) are indistinguishable from wild‐type mice following *M. tuberculosis* infection, except for a reduction in splenic bacterial burden.^23^ However, IFNAR deletion in more susceptible mouse strains substantially improves survival, with mice having much lower bacterial burdens.^24^, ^25^ These data from murine models, together with evidence from humans,^26^ support the notion that type I IFNs are associated with TB disease progression. The mechanism for this remains unclear, but recent work suggests that the inhibition of host‐protective cytokines (TNF, IL‐12, IL‐1β), blunting of IFN‐γ responsiveness, and induction of immunosuppressive IL‐10 may be involved.^25^, ^27^ In fact, Wassermann and colleagues^22^ showed that secretion of IL‐10 was reduced in infected cGAS‐ and STING‐deficient macrophages. They further demonstrated that macrophages infected with the hypervirulent HN878 strain, which induces a stronger type I IFN response, benefited from cGAS or STING deletion in terms of reduced IL‐10 production and enhanced survival. It would be interesting to examine the outcome of infection of *Cgas^−/−^* mice with HN878, and indeed, cGAS deficiency in a more susceptible mouse strain.

IFN‐β production following *M. tuberculosis* infection is dependent on a functional ESX‐1 secretion system,^20^, ^22^, ^23^ which indicates that the mycobacterial DNA that activates cGAS originates from living rather than dead/degrading bacteria. Although it remains unclear exactly how *M. tuberculosis* releases its DNA into the cytosol, it is tempting to speculate that this occurs deliberately in order to coopt host signaling in favor of IFN‐β production. However, subsequent work showed that strain‐dependent differences in IFN‐β production were at least partially related to the level of mitochondrial stress and mitochondrial DNA released into the cytosol due to *M. tuberculosis* infection, and not due to variability in bacterial access to the cytosol or shedding of bacterial DNA.^28^ The detection of host mitochondrial DNA by cGAS may thus contribute to type I IFN production. It remains unclear exactly why strains vary in their ability to induce mitochondrial stress/DNA release, but is most likely due to variability in the expression of particular virulence factors. It is almost certain, however, that additional factors or pathways contribute to differences in IFN‐β production between strains, as mitochondrial involvement does not completely account for these differences.^28^

### 1.4 Short‐circuiting signal transduction pathways

Macrophages infected with *M. tuberculosis* produce a number of cytokines in order to orchestrate an effective immune response to the pathogen. The synthesis of many host‐protective cytokines is regulated by the NF‐κB and MAPK signaling pathways. Given that several such cytokines, such as TNF, IL‐1β, and IL‐6, are potent, nonredundant mediators of anti‐TB immunity, it is perhaps not surprising that their regulatory pathways represent attractive targets for dampening the host immune response to infection. For instance, the *M. tuberculosis* virulence protein PtpA inhibits the JNK, p38, and NF‐κB pathways in macrophages (Fig. 1).^29^ The inhibition of the JNK and p38 MAPK pathways is dependent on the phosphatase activity of PtpA, which dephosphorylates phospho‐JNK and phospho‐p38. Interestingly, this activity is itself stimulated by the binding of ubiquitin‐interacting motif‐like region of PtpA to host ubiquitin, which may serve to restrain its phosphatase activity until the protein is secreted into the cytosol. PtpA also competitively binds to TAB3, blocking its ability to bind to K63 ubiquitin chains and thereby partially interfering with NF‐κB activation. A subsequent study identified the RING domain of host TRIM27 as an additional target of PtpA.^30^ TRIM27 has E3 ubiquitin ligase activity and promotes JNK/p38 pathway activation to restrict *M. tuberculosis*. By deleting *PtpA* from *Mycobacterium bovis* bacillus Calmette‐Guérin (BCG), these studies showed that the protein suppresses the production of TNF, IL‐1β, and IL‐12 by macrophages both *in vitro* as well as in mice, which also harbored fewer bacteria in the lungs compared to mice infected with wild‐type BCG.^29^, ^30^ This observation contrasts with the deletion of *PtpA* in *M. tuberculosis* that, as discussed earlier, does not attenuate the pathogen during infection *in vivo*.^9^ The discrepancy is most likely explained by the fact that *M. tuberculosis* possesses many additional virulence factors (such as ESX‐1) that BCG lacks, and that may render the function of PtpA redundant *in vivo*.

The ability of PtpA to inhibit cytokine production appears to be mostly due to its impairment of NF‐κB signaling, with contribution from its MAPK‐inhibitory activity, particularly JNK and p38, but not ERK1/2.^29^ Other virulence factors of *M. tuberculosis* have also been reported to interfere with NF‐κB activation. For example, treatment of cells with purified early secreted antigenic target 6 (ESAT‐6)—the major ESX‐1 substrate—inhibits NF‐κB activation downstream of TLRs, thereby attenuating TNF and IL‐6 release.^31^ Interestingly, ESAT‐6 is capable of preventing TLR‐mediated NF‐κB activation downstream of all TLRs, despite only binding directly to TLR2. This binding activates cytosolic Akt kinase that appears to prevent the formation of MyD88 signaling complexes, and therefore NF‐κB activation, following ligation of other TLRs. The mammalian cell entry protein 3E (Mce3E), which is encoded by the *mce3* operon, is secreted by phagocytosed *M. tuberculosis* and is expressed during infection in humans.^32^, ^33^ MceE3 was recently shown to interfere with the ERK1/2 MAPK signaling pathway by entering the cytosol and localizing to the endoplasmic reticulum (ER).^33^ Here, it interacts with ERK1/2, tethering it to ER. This serves to block both the phosphorylation of ERK1/2 by MEK1, as well as the nuclear translocation of phopho‐ERK1/2. Expression of Mce3E in *Mycobacterium smegmatis* and BCG downregulated TNF and IL‐6 production by infected macrophages and promoted intracellular growth. However, deletion of the entire *mce3* operon in *M. tuberculosis* reportedly did not affect cytokine induction or intracellular survival in macrophages.^34^ Although lung bacterial burdens in mice were also unaltered by infection with the *mce3*‐deleted strain, mice survived slightly longer than those infected with wild‐type *M. tuberculosis*.

Collectively, these studies reveal novel mechanisms by which *M. tuberculosis* may interfere with several major host signal transduction pathways, and thereby modulate cytokine production to the detriment of the host. Further work will be required to elucidate the *in vivo* contributions of the various virulence factors that have been implicated.

### 1.5 Metabolic reprogramming of host cells

The metabolic versatility of *M. tuberculosis* enables it to grow on a variety of carbon sources, and the available evidence indicates that during intracellular growth, the pathogen relies primarily on cholesterol ester and fatty acid metabolism.^35^, ^36^, ^37^ *Mycobacterium tuberculosis* adjusts macrophage metabolism under hypoxic conditions to promote the accumulation of lipid bodies, giving rise to the “foamy” macrophages characteristically found at the interface of central necrotic regions within granulomas.^38^ *Mycobacterium tuberculosis* laden phagosomes have been shown to interact with, and release bacilli into, host lipid bodies, which serve as a critical source of nutrients in an otherwise nutritionally devoid phagosome.^38^ This interaction with host lipid bodies also generates a secure niche within which the pathogen is protected from bactericidal mechanisms such as respiratory burst. Additionally, mycobacteria contained within lipid bodies acquire a dormancy phenotype, which confers tolerance to several front‐line antibiotics.^39^

**Figure 2:**
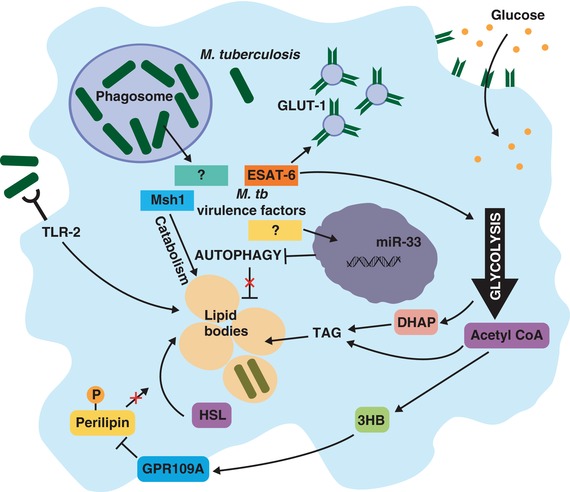
***Mycobacterium tuberculosis* alters host cell metabolism to promote “foamy” macrophages containing lipid bodies**. Mycobacterial cell wall components can stimulate lipid body accumulation in a partially TLR2‐dependent manner. The virulence factor ESAT‐6 also promotes glycolytic flux by stimulating the translocation of GLUT‐1 glucose transporters to the cell surface to enhance glucose uptake, and by directly modulating the activity of key glycolytic enzymes. This promotes the accumulation of dihydroxyacetone phosphate (DHAP) and acetyl CoA, which can both be used as substrates for triacylglycerol (TAG) synthesis. Acetyl CoA is also converted into D‐3‐hydroxybutyrate (3HB), which activates the G protein coupled receptor GPR109A. This inhibits the phosphorylation of perilipin, thereby restricting the translocation of hormone‐sensitive lipase (HSL) to lipid bodies, and preventing their degradation. *Mycobacterium tuberculosis* also inhibits autophagic degradation of lipid bodies by inducing miR‐33 expression. The pathogen can reside within these lipid bodies and acquire a protective dormancy phenotype, or can utilize the lipids as a source of nutrients by secreting the hydrolytic Msh1 protein

Although the advantages that lipid body accumulation confers to *M. tuberculosis* are well established, insights into the mechanisms by which the pathogen coopts the macrophage to induce the foamy phenotype have only recently come to light (Fig. 2). Macrophages are driven into an anabolic state by ESAT‐6, which stimulates the translocation of GLUT‐1 glucose transporters from the cytosol to the cell membrane, thereby drastically enhancing glucose uptake and metabolism.^40^, ^41^ ESAT‐6 also appears to heighten the activity of several glycolytic enzymes, thus perturbing the normal flux between glycolysis and the tricarboxylic acid cycle. This leads to the accumulation of dihydroxyacetone phosphate, which is used as a substrate for the synthesis of triacylglycerol (TAG).^40^ Concurrently, intracellular concentrations of acetyl CoA also rise, which promotes ketogenesis and results in shunting of acetyl CoA toward the synthesis of the ketone body d‐3‐hydroxybutyrate (3HB),^40^, ^42^ although some of this acetyl CoA may also be directed toward de novo lipid synthesis.^41^ Secreted 3HB activates the antilipolytic G protein coupled receptor GPR109A, which inhibits the pathway leading to the phosphorylation of perilipin.^42^ The absence of phosphorylated perilipin prohibits the translocation of hormone‐sensitive lipase to lipid bodies, thereby preventing the mobilization of stored TAG. Thus, ESAT‐6 perturbs host glucose metabolism to both promote the accumulation of lipid bodies and therefore the differentiation of “foamy” macrophages, and simultaneously arrest lipid catabolism. However, this enhancement of glycolysis also induces IL‐1β and suppresses IL‐10, which is required for controlling intracellular replication.^43^ Additionally, studies showed that lipid body formation does not require live bacilli and can be driven by mycobacterial cell wall components such as lipoarabinomannan and trehalose dimycolate, in a partially TLR2‐dependent manner.^44^, ^45^, ^46^ This would suggest that modulation of glycolysis by ESAT‐6 may not represent the sole or major driving force of “foamy” macrophages. Ouimet et al. recently demonstrated that infection of macrophages, *in vitro* and in mice, with *M. tuberculosis* caused upregulation of the host microRNA miR‐33.^46^ The elevated levels of miR‐33 were found to repress key effectors of autophagy—a cellular process of degrading intracellular components by engulfing them within membrane‐bound vesicles for delivery to lysosomes. Autophagy is known to promote lipid catabolism (lipophagy) by degrading TAG and cholesterol esters within lipid bodies.^47^ Not surprisingly then, miR‐33‐mediated repression of autophagy caused a reduction in cellular fatty acid oxidation and bolstered the size and number of lipid bodies, which supports the metabolism and growth of intracellular mycobacteria.^46^ Consistent with previous reports, dead mycobacteria and purified cell wall constituents were sufficient to induce miR‐33 expression in an NF‐κB‐dependent manner. Mice reconstituted with *Mir33^−/−^* hematopoietic cells had slightly lower burdens of *M. tuberculosis* in the lungs, demonstrating that miR‐33 induction may contribute to disease *in vivo* by limiting autophagy and lipid catabolism, thereby promoting lipid body accumulation.

Several studies have demonstrated that host fatty acids are incorporated into intracellular *M. tuberculosis* and either stored as TAG or used as a source of nutrients.^39^, ^46^ However, little attention has been given to how the pathogen mobilizes host TAG stores in order to import the fatty acids into the bacteria. Recent work showed that *M. tuberculosis* secretes a membrane‐associated hydrolytic protein (Msh1) into macrophages under hypoxic stress.^48^ Msh1 is expressed during infection *in vivo*, and functions to catalyze host lipid hydrolysis. Thus, *M. tuberculosis* has devised strategies to both stockpile essential nutrients in the form of lipid bodies, as well as to tap into these reserves as required, perhaps during reactivation from latency.

### 1.6 Inhibition of autophagy and antigen presentation

**Figure 3:**
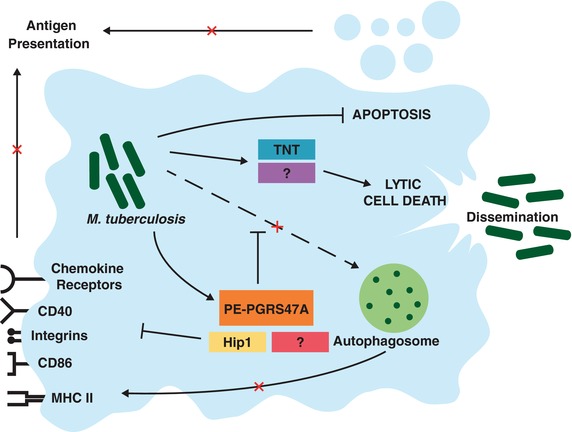
***Mycobacterium tuberculosis* modulates the macrophage death modality to promote dissemination and interfere with adaptive immunity**. PE‐PGRS47A obstructs autophagosomal processing of mycobacteria, and therefore the presentation of *M. tuberculosis* peptides by MHC class II. Other virulence factors reduce the expression of cell surface molecules required for antigen presentation. Through a variety of reported mechanisms, *M. tuberculosis* also inhibits macrophage apoptosis, a process which would otherwise result in mycobacterial killing and facilitate priming of adaptive immunity. The pathogen exits the host cell and disseminates by inducing a lytic form of death. The processes leading to this are poorly understood and may involve secretion of tuberculosis necrotizing toxin (TNT)

It is prudent to highlight the long‐standing controversy surrounding the role of autophagy in *M. tuberculosis* disease pathogenesis. While initially thought to constitute an essential host defensive strategy whereby the macrophage encloses intracellular mycobacteria in membranes for lysosomal degradation,^49^, ^50^, ^51^ Kimmey et al. used a sleuth of gene‐targeted mice to convincingly demonstrate the absence of an *in vivo* role of autophagy in the control of *M. tuberculosis*.^52^ However, this does not exclude the possibility that *M. tuberculosis* inhibits the autophagic process (Fig. 3). This notion is supported by several reports implicating various mycobacterial virulence factors as mediators of this inhibition.^53^, ^54^, ^55^ Besides promoting the accumulation of nutrients, blocking autophagic processes is believed to benefit intracellular pathogens including *M. tuberculosis* by limiting the presentation of its antigens to T cells by MHC class II.^56^, ^57^, ^58^ The *M. tuberculosis* protein PE_PGRS47A was recently identified in a genome‐wide screen for genetic loci of *M. tuberculosis* that interfere with MHC class II restricted antigen presentation.^58^ *Mycobacterium tuberculosis* secretes PE‐PGRS47A into the cytosol of infected macrophages and dendritic cells (DCs), where it prevents the formation of autophagosomes containing intracellular bacilli. Infection of DCs *in vitro* with a PE‐PGRS47A deletion mutant enhanced their capacity to present *M. tuberculosis* antigens to T cells and also reduced intracellular bacterial numbers. In agreement with this, mice infected with the mutant strain generated a significantly stronger *M. tuberculosis* specific CD4^+^ T‐cell response that, at chronic stages of infection, substantially attenuated bacterial growth and tissue damage in all organs. However, severe combined immunodeficient mice, which are incapable of mounting adaptive immune responses, survived longer when infected with the deletion mutant, indicating that PE‐PGRS47A also interferes with innate immunity. Thus, by inhibiting autophagy, *M. tuberculosis* is able to efficiently modulate both innate killing as well as adaptive immunity.

Besides interfering with autophagy, *M. tuberculosis* has been reported in *in vitro* studies to manipulate antigen presentation by several other means. For example, the latency‐associated protein Acr1 was found to impair the maturation of DCs.^59^ Additionally, the mycobacterial serine hydrolase Hip1 reduced surface expression of MHC class II and the costimulatory molecules CD40 and CD86, as well as a number of pro‐inflammatory cytokines.^60^ Several groups have also reported that infection of DCs with *M. tuberculosis* reduces their expression integrins and chemokine receptors,^61^, ^62^ therefore hindering their migration to lymph nodes and delaying the onset of adaptive immunity.^63^, ^64^ It is also thought that the cross‐presentation of *M. tuberculosis* antigens is prevented by the inhibition of apoptosis of infected phagocytes,^65^ as discussed below.

### 1.7 Directing the macrophage death modality

Failure to eliminate intracellular *M. tuberculosis* inevitably leads to death of the infected phagocyte. However, the manner in which *M. tuberculosis* infected macrophages die remains enormously controversial. A substantial body of research supports the prevailing opinion that virulent *M. tuberculosis* limits apoptosis of the cells they infect, instead promoting a lytic or necrotic form of death (Fig. 3).^66^, ^67^, ^68^, ^69^, ^70^, ^71^, ^72^, ^73^ Apoptosis is thought to result in the killing of intracellular mycobacteria and the priming of adaptive immunity when apoptotic bodies of infected cells are engulfed and degraded by uninfected macrophages in a process termed efferocytosis.^65^, ^74^, ^75^, ^76^, ^77^ The finding that avirulent strains of *M. tuberculosis* primarily induce apoptosis, while virulent strains induce mostly a lytic death modality is in keeping with the notion that apoptosis is protective for the host, and is thus a target for virulent mycobacteria. Multiple proteins secreted by *M. tuberculosis* into the macrophage cytoplasm have been reported to inhibit apoptosis by diminishing the availability of caspase 8,^71^ interfering with apoptotic envelope formation,^68^ dephosphorylating GSK3α,^78^ inactivating TNF by inducing release of TNF receptor 2^67^ and inducing antiapoptotic Mcl‐1,^69^ as well as other proteins for which the mechanisms have not been elucidated.^79^, ^80^ However, some groups maintain that virulent *M. tuberculosis* exclusively induces apoptosis.^81^, ^82^, ^83^ This discrepancy is difficult to reconcile, but is likely due to differences in the experimental systems used in these studies, especially in relation to the species of origin and mortality of the cells being used.

While it is clear that a small proportion of macrophages infected even with virulent *M. tuberculosis* die by apoptosis,^76^ the majority succumb to pathogen‐induced lytic death. This death modality is believed to allow *M. tuberculosis* to evade host immunity, disseminate to neighboring cells, breach the airways, and ultimately transmit to new hosts in order to complete its pathogenic life cycle.^73^, ^84^ The detailed mechanism by which *M. tuberculosis* achieves this is unclear, but may relate to cell membrane microdisruptions caused by the pathogen, and the simultaneous induction of lipoxin A_4_, which blocks prostaglandin E_2_‐mediated membrane repair.^65^, ^85^ Recent work also showed that *M. tuberculosis* secretes tuberculosis necrotizing toxin (TNT) into the macrophage cytosol, which hydrolyzes the coenzyme NAD^+^.^86^ Depletion of NAD^+^ results in macrophage necrosis, although the downstream events leading to this remain undefined. Intriguingly, *M. tuberculosis* produces an immunity factor for TNT (IFT), which protects mycobacterial cells from TNT‐mediated toxicity by inhibiting its activity.^86^ It is tempting to speculate that TNT secretion may enable the pathogen to fine‐tune the timing of macrophage death. Collectively, the literature supports a model whereby *M. tuberculosis* dictates the mode, and most likely the timing, of death of their host cells, in order to prevent mycobacterial killing, blunt the immune response, and disseminate.

## 2 CONCLUSIONS

Given the enormous body of literature concerning the manipulation of host signaling by *M. tuberculosis*, we have attempted to summarize the most recent gains in our understanding of host‐pathogen interactions in TB. The pathogen has at its disposal an assortment of virulence factors that display incredible breadth in terms of host cellular targets. It is pertinent to bear in mind, however, that the human host is by no means a defenseless counterpart in the pathogenic process. On the contrary, we possess a potent repertoire of countermeasures that, in the majority of individuals, are reasonably successful in at least containing the infection. It is interesting to note that the individual deletion of many of the virulence factors of *M. tuberculosis* causes only a mild or no effect on disease *in vivo*. This provides qualified support for the notion that many of these virulence factors play redundant roles during infection, perhaps because they target the same host signaling pathways. Speculatively, this redundancy may have evolved in response to the multifaceted defense systems of the host. Hence, deciphering how *M. tuberculosis* thwarts host signaling to cause disease is imperative, not only in paving the way for novel therapeutic targets, but perhaps in beginning to understand why some individuals are more susceptible to active TB disease than others.
